# Protection of Vitamin C on Oxidative Damage Caused by Long-Term Excess Iodine Exposure in Wistar Rats

**DOI:** 10.3390/nu14245245

**Published:** 2022-12-09

**Authors:** Rong Sun, Lanchun Liu, Tingting Qian, Meng Zhao, Wenjing Che, Xin Hou, Honglei Xie, Yue Su, Haowen Pan, Jia Li, Peng Liu

**Affiliations:** 1 Center for Endemic Disease Control, Chinese Center for Disease Control and Prevention, Harbin Medical University, Harbin 150081, China; 2 Key Lab of Etiology and Epidemiology, Education Bureau of Heilongjiang Province (23618504), Ministry of Health, Microelement and Human Health Lab of Heilongjiang Province, Harbin 150081, China

**Keywords:** vitamin C, iodine excess, protection, oxidative damage

## Abstract

Vitamin C was reported to be able to protect against oxidative damage due to its reducibility. 120 Wistar rats were randomly divided into 4 × 2 groups, including normal iodine (NI), high iodine (HI), low vitamin C (HI + LC), and high vitamin C (HI + HC); potassium iodide (KI) and potassium iodate (KIO_3_) were commonly used as additives for iodized salt, so every group was also divided into KI and KIO_3_ groups. After 6 months’ feed, the activities of antioxidant enzymes and Lipid Peroxide (MDA) content in serum, liver, kidney, brain, thyroid and lens were determined. In serum, for males, long-term excess iodine intake caused oxidative damage; in the liver, male rats in the HI + LC group had the highest MDA content, which showed that low-dose vitamin C might promote oxidative damage; in kidneys, the MDA content in the HI and HI + LC groups of females was higher; in the brain, high-dose vitamin C could increase the activity of superoxide dismutase (SOD), which was decreased by high iodine intake, and it also decreased MDA content; in the thyroid, for KIO_3_, the activity of SOD in the HI group was lower than NI and HI + LC; in the lens, the MDA content in females was lower than males. Long-term excess iodine exposure caused oxidative damage and showed sex difference, and vitamin C had a protective effect on it, especially for high-dose vitamin C.

## 1. Introduction

As an essential trace element, iodine (I) acts on many organs of the body through the synthesis of thyroid hormones to promote biological oxidation, regulate the metabolic level, and promote growth [1] and development [2]. Inadequate iodine intake can lead to iodine deficiency disorders (IDD) represented by endemic goiter and endemic cretinism [3]. As a global recommendation strategy, the universal salt iodization has been implemented to prevent and control IDD in many countries for many years [4] (pp. 641–647). In China, it was implemented in 1995, and IDD had been eliminated in 28 out of 31 provinces by the year 2010 [5]. However, the epidemic spectrum and magnitude of thyroid diseases have changed, such as thyroid dysfunction [6], autoimmune thyroid disease [7], nodules and thyroid cancer [8], etc. Therefore, experts and scholars have carried out a series of studies on the safety of iodine intake.

Bürgi et al. reviewed more than 50 literature sources from 1941 to 2000, and elaborated on the safety of potassium iodate (KIO_3_) as a salt-fortified iodine preparation [9]. As the main iodized salt fortifier, potassium iodide (KI) and KIO_3_ had different chemical properties and biological effects [10]. KIO_3_ was more stable and easier to store than KI, but it belonged to halogen oxygenates, which could easily obtain electrons and be reduced to I-, so KIO_3_ had strong oxidizability and might cause oxidative damage to the body. In addition, studies have shown that iodine excess posed a direct threat to thyroid cells, increased the production of reactive oxygen species (ROS), broke the balance between oxidants and antioxidants, and caused oxidative stress [11,12], which would decrease the activities of antioxidant enzymes and cause oxidative damage in serum, the liver, the kidney, the brain, the thyroid, and the lens and further cause autoimmune thyroid disease, lipid metabolism disorders, etc. [13].

Legislation stipulates salt iodine fortification above the recommended level or excessive salt iodization and poor monitoring at production are a common cause of excessive iodine exposure in various countries [14,15], such as in Cameroon, Honduras, and Colombia, where their iodine content in salt was 100, 83, and 75 mg/kg, respectively. Moreover, the health hazards of high iodine consumption, derived from the geological structure, diet, and the improper use of iodized salt and iodine oil capsules, have become increasingly prominent [16]. As has been estimated, the number of iodine-excessive countries had risen to 14 in 2021, relative to 5 in 2003. In China, about 61 counties were areas with high water iodine (I^−^ and IO_3_^−^) [17]; people in these areas were extremely vulnerable to excessive intake of iodine (including potassium iodate) [18]. An epidemiological study observed chronic exposure to high water iodine was associated with primary hypothyroidism (PH) and subclinical hypothyroidism (SCH) [19]. As a result, it becomes important to explore additional supplements to organisms for counteracting the oxidative damage imposed by excess iodine exposure.

Vitamin C (C_6_H_8_O_6_), a common reducing substance in food, is a member of the non-enzymatic antioxidant system for the human body. It forms a reversible redox system with ascorbic acid–dehydroascorbic acid, which plays an important role in biological oxidation and reduction [20]. It is an active oxygen derivative and free radical scavenger, inactivating agent, and regenerates other antioxidants in the body, such as α-tocopherol (vitamin E) [21]. Moreover, studies have shown that vitamin C could stimulate the formation of bile in the gallbladder, promote the excretion of cholesterol and steroids, and effectively prevent lipid peroxidation caused by peroxide free radicals [22,23].

In recent years, there have been many studies on the protection of oxidative damage caused by excessive iodine. Ji SS et al. found that procyanidins could significantly improve the antioxidant capacity and protect the normal morphology of thyroid cells, thus protecting the thyroid from oxidative damage caused by KIO_3_ [24], and one study illustrated that melatonin, a safe and strong antioxidant, should be considered as a potential protective agent against oxidative damage to membrane lipids caused by KIO_3_ [25]. Zhang YP et al. carried out an experimental study on the scavenging of iodate ions (IO_3_^−^) by vitamin C under the condition of simulated gastric juice in vitro; the conversion rates of IO_3_^−^ to I^−^ at different concentrations, acidity, and reaction time by vitamin C were determined, and the results showed that vitamin C could reduce IO_3_^−^ to I^−^ rapidly and quantified it according to the chemical equation [26]. However, these studies were aimed at the protection from oxidative damage caused by excessive KIO_3_ with ignoring excessive KI, and Zhang YP’s research was carried out in vitro; there is a lack of experimental information in vivo.

This study was carried out for the following purposes: first, to study whether long-term chronic excessive iodine intake will lead to a decrease in antioxidant enzyme activity and lipid peroxidation damage; secondly, to study whether KI and KIO_3_ have different effects on oxidative damage; and finally, to simulate the protective effect of oxidative damage caused by excess iodine exposure with additional vitamin C intake through vegetables, fruits, etc., and whether there are sex differences.

## 2. Materials and Methods

### 2.1. Reagents

KI and KIO_3_ were standard reagents purchased from Shanghai Yindian Chemical Co., Ltd., Shanghai, China, and Tianjin Yongda Chemical Reagent Co., Ltd., Tianjin, China, respectively.

### 2.2. Dose Calculation

In the current study, the oxidative damage of long-term excess iodine exposure and its amelioration by vitamin C was evaluated via the activity of antioxidant enzymes and lipid peroxide content. For the dose of vitamin C used in this study, the recommended intake and tolerable maximum intake of vitamin C in the human body were referenced [27], based on the 60 kg of normal adult body weight, and using a certain conversion coefficient (36) as the tolerance of animals, the experimental doses were calculated to be 60 μg/g.bw/d and 600 μg/g.bw/d, respectively. Then, it was found that the conversion coefficient between daily drug intake and diet was 20 [28], and the content of vitamin C in the diet was calculated (1.2 g/kg and 12 g/kg).

### 2.3. Animal and Treatments

120 Wistar rats (SPF, 120–160 g), purchased from Beijing Weitong Lihua Experimental Animal Technology Co., Ltd., Beijing, China, were randomly divided into 4 × 2 groups according to their body weight, with 10 female and 5 male rats in each group: two groups of optimal iodine (NI), fed deionized water containing 50 μg/L iodine ion with KI or KIO_3_, separately (NKI and NKIO_3_); two groups of high iodine (HI), fed deionized water containing 50 mg/L iodine ion (HKI and HKIO_3_); two groups of high iodine and low vitamin C (HI + LC), fed 50 mg/L iodine water and diet with 1.2 g/kg vitamin C (HKI + LC and HKIO_3_ + LC); and two groups of high iodine and high vitamin C (HI + HC), fed 50 mg/L iodine water and diet with 12 g/kg vitamin C (HKI + HC and HKIO_3_ + HC). NI and HI were fed a maintenance diet (no vitamin C in feed ingredients). All diets were purchased from Beijing Keao Xieli Feed Co., Ltd., Beijing, China. Wistar rats were kept in the animal room with ambient temperature 20 ± 2 °C, relative humidity between 40% and 80%, with 12 h alternate light or dark cycles, respectively, and drank and ate freely. All animals were intervened for 6 months.

### 2.4. Samples Collection

During the feeding process, the rats’ weekly drinking water and diet intake were recorded with the cage as a unit (five rats). The drinking water was measured with a measuring cylinder and the diet was weighed with a balance. One week before killing the rats, urine samples were collected in a metabolic cage for the determination of urinary iodine. The rats were anesthetized by an intraperitoneal injection of 10% chloral hydrate. After the rats were anesthetized completely, 5 mL blood was collected in the vessels without anticoagulants from the abdominal aorta and placed for 2 h, then 3000 rpm/min centrifugation for 10 min; the upper serum was packed and stored in the refrigerator at −80 °C. In addition, after the rats were sacrificed; their livers, kidneys, brains, thyroids, and lenses were surgically removed and preserved in liquid nitrogen, and then transferred to the refrigerator at −80 °C for storage.

### 2.5. Preparation of Tissue Homogenate

Firstly, the livers, kidneys, brains, thyroids, and lenses were accurately weighed; then, according to the weight (g) and volume (ml) ratio of 1:9, we added 9 times the volume of physiological saline; finally, under the conditions of an icy water bath, 10% homogenate was prepared, centrifuged at 2500 rpm/min for 10 min, and the supernatant was taken to be determined. The protein content of homogenate was determined with coomassie brilliant blue protein (purchased from Nanjing Jiancheng Technology Co., Ltd., Nanjing, China).

### 2.6. Determination of Antioxidant Enzymes Activity and MDA Content

Total antioxidant capacity (TAOC) was determined using the ferric reducing ability of plasma method (FRAP) [29]. Catalase (CAT) was determined using the ammonium molybdate method, which was that the decomposition of hydrogen peroxide (H_2_O_2_) by CAT could be quickly terminated by the addition of ammonium molybdate, and the remaining H_2_O_2_ reacted with ammonium molybdate to produce a yellowish complex; then, the variation was measured at 405 nm using a microplate reader (produced by Bio-Rad Instrument Corporation of America) and the activity of CAT could be calculated. Superoxide dismutase (SOD) was determined using the hydroxylamine method [30], the content of the Malondialdehyde (MDA) was determined using the TBA method [31], and the glutathione peroxidase (GSH-Px) activity was determined by measuring the reaction rate of glutathione and hydrogen peroxide [32]. All the kits were purchased from Nanjing Jiancheng Technology Co., Ltd., Nanjing, China. In addition, the optical density (OD) value was read by a microplate reader, and two parallel samples were used to ensure the accuracy of the experiment.

### 2.7. Determination of Urinary Iodine

Arsenic and cerium catalytic spectrophotometry were used to determine urine iodine (WS/T107.1-2016).

### 2.8. Statistical Analysis

The SPSS 22.0 (produced by International Business Machines Corp, Aromnk, NY, USA) was used for statistical analysis. All the data are presented as mean and standard deviation, but the urinary iodine is presented as median and interquartile. For normal distribution data, two independent samples *t* test and variance analysis (ANOVA) were used to compare the activities of antioxidant enzymes and the content of MDA in different groups and between females and males. Moreover, a Tukey test was used to conduct a post hoc test; for skewed distribution data, a Kruskal–Wallis test and Mann–Whitney test were used to compare the activities of antioxidant enzymes and the content of MDA in different groups. All tests were two-sided and *p* < 0.05 was defined as significant.

## 3. Results

### 3.1. Water and Food Consumption and Iodine Intake

Comparing the amount of drinking water in each group in Table S1, it was found that the amount of drinking water in each group was relatively balanced, but sometimes, the amount of drinking water of the low-vitamin-C group with KIO_3_ was lower than that of other groups (both female and male rats), which was the same for female rats in potassium iodide. In addition, according to the food consumption of each group in Table S2, the rats in the low-vitamin-C group generally ate less; however, the food consumption of rats in the high-vitamin-C group was more than the optimal-iodine group and high-iodine group. Moreover, the daily iodine intake of rats was calculated according to the consumption of water and the diet. Whether different sex or different iodine type, the iodine consumptions of rats in the HI, HI + LC, and HI + HC groups were higher than that in the NI group.

### 3.2. Urinary Iodine

Whether KI or KIO_3_, compared with NI, Table 1, the urinary iodine level of rats in the HI, HI + LC, and HI + HC groups increased significantly (*p* < 0.01), which was the same as the different sex. However, no statistically significant changes were found in the HI, HI + LC, and HI + HC groups by the Kruskal–Wallis test, which indicated that the intake of vitamin C had no effect on iodine excretion in rats.

### 3.3. Protection of Vitamin C from Oxidative Damage in Serum Caused by Excess Iodine

**Figure 1:**
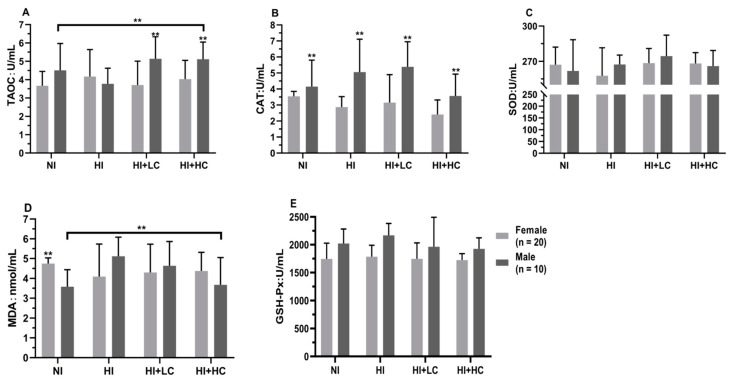
Protection of vitamin C from oxidative damage in serum for different sexes. (**A**) The activity of TAOC; (**B**) The activity of CAT; (**C**) The activity of SOD; (**D**) The content of MDA; (**E**) The activity of GSH-Px. All data are presented as mean and deviation. ** represents *p* < 0.01.

First of all, from the perspective of different sources of iodine, Table S3, compared with NI, there were no statistical differences in the activities of all antioxidant enzymes and MDA content in the serum of HKI and HKIO_3_, but the activity of SOD in HKI was significantly higher than that in HKIO_3_ (*U* = −2.281, *n* = 15, *p* = 0.005). Then, in terms of female rats, Figure 1, there was no significant change compared with NI; moreover, as for male rats, high iodine intake significantly decreased the activity of TAOC (*F* = 3.254, *df* = 39, *p* = 0.033) and increased the content of MDA (HI and NI, *U* = −2.859, *n* = 10, *p* = 0.003; NI and HI + LC, *U* = −2.330, *n* = 10, *p =* 0.017), which showed that long-term, high iodine intake would cause the decompensation of antioxidant enzyme activity and oxidative damage, but the intake of vitamin C would block the process. Finally, there were differences between females and males, such as the CAT activity of female rats being significantly lower than that of male rats (*U* = −5.360, *n* = 10, *p* = 0.002).

### 3.4. Protection of Vitamin C from Oxidative Damage in Liver Caused by Excess Iodine

**Figure 2:**
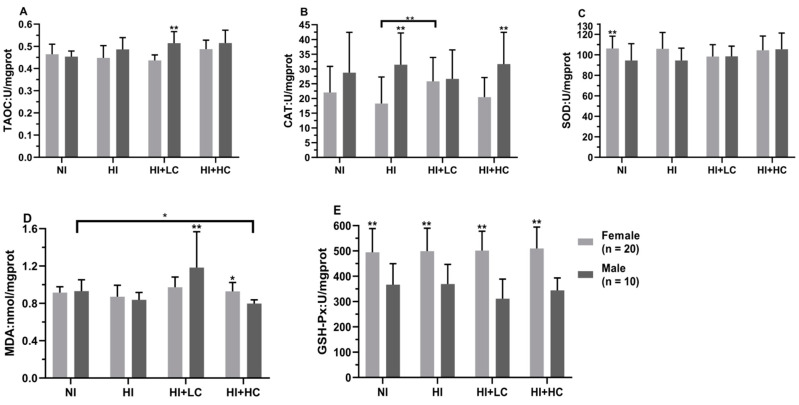
Protection of vitamin C from oxidative damage in liver for different sexes. (**A**) The activity of TAOC; (**B**) The activity of CAT; (**C**) The activity of SOD; (**D**) The content of MDA; (**E**) The activity of GSH-Px. All data are presented as mean and standard deviation. ** represents *p* < 0.01, and * represents *p* < 0.05.

In Table 2, as for the different iodine sources, the MDA content in the HI + LC group was higher than the other groups, but only KI had statistical significance (*H* = 9.076, *df* = 3, *p* = 0.023); in addition, the activities of TAOC, CAT, SOD, and GSH-Px had no significant changes in KI and KIO_3_. In addition, the male rats in the HI + LC group were decompensated with the highest MDA content (*H* = 17.925, *df* = 3, *p* = 0.000), Figure 2. In the same group, the content of MDA in females was lower than that in males (*U* = −3.388, *n* = 10, *p* = 0.000), which was opposite to that in the HI + HC group, and compared with female rats in HI, the activity of CAT in the HI + LC group was significantly higher (*U* = −2.776, *n* = 20, *p* = 0.008). Finally, in terms of different sexes, the CAT activity of females was significantly lower than males in the HI (*t* = −3.536, *df* = 39, *p* = 0.001) and HI + HC (*t* = −3.528, *df* = 39, *p* = 0.001) groups, which was similar in the NI and HI + LC groups, but the difference was not statistically significant, and the activity of GSH-Px in females was significantly higher than that in males (*t* = 9.709, *df* = 29, *p* = 0.000).

### 3.5. Protection of Vitamin C from Oxidative Damage in Kidney Caused by Excess Iodine

**Figure 3:**
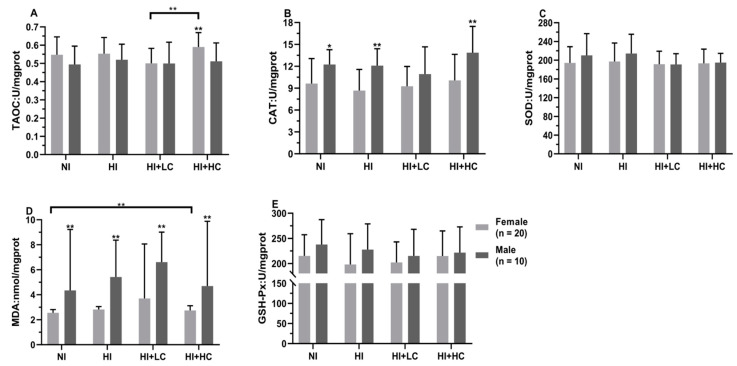
Protection of vitamin C from oxidative damage in the kidney for different sexes. (**A**) The activity of TAOC; (**B**) The activity of CAT; (**C**): The activity of SOD; (**D**) The content of MDA; (**E**) The activity of GSH-Px. All data are presented as mean and deviation. ** represents *p* < 0.01, and * represents *p* < 0.05.

There was no significant difference in the activities of antioxidant enzymes among every group in KI and KIO_3_, Table 3, but low-dose vitamin C had the highest level of lipid peroxidation (KI, *H* = 10.456, *df* = 3, *p* = 0.015 and KIO_3_, *H* = 12.974, *df* = 3, *p* = 0.005). Moreover, as for female rats in the HI + LC group, the TAOC activity was lower than the HI + HC (*t* = −3.553, *df* = 29, *p* = 0.001) group, Figure 3, and its MDA content was significantly higher than other groups (*H* = 30.861, *df* = 3, *p* = 0.001), while all indicators of male rats had no statistical significance. Finally, in terms of the comparison between female and male, it was found that the female MDA content was significantly lower than male (*U* = −5.597, *n* = 10, *p* = 0.000), and it was similar to the CAT activity in the NI (*t* = −2.213, *df* = 29, *p* = 0.036), HI (*t* = −3.246, *df* = 29, *p* = 0.003) and HI + HC (*t* = −2.742, *df* = 29, *p* = 0.011) groups.

### 3.6. Protection of Vitamin C from Oxidative Damage in the Brain Caused by Excess Iodine

**Figure 4:**
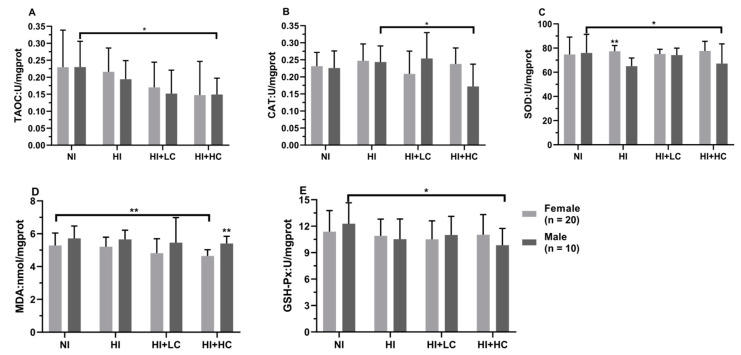
Protection of vitamin C from oxidative damage in the brain for different sexes. (**A**) The activity of TAOC; (**B**) The activity of CAT; (**C**) The activity of SOD; (**D**) The content of MDA; (**E**) The activity of GSH-Px. All data are presented as mean and deviation. ** represents *p* < 0.01, and * represents *p* < 0.05.

Firstly, either for KI or KIO_3_, different intervention measures had no significant effect on the antioxidant enzyme activity and MDA content in each group, Table S4, nor was there a difference between KI and KIO_3_. Secondly, as for female rats, high-dose vitamin C decreased the content of MDA and had statistical significance (NI and HI + HC, *U* = −2.516, *n* = 20, *p* = 0.011; HI and HI + HC, *U* = −2.029, *n* = 20, *p* = 0.043), Figure 4, but the activity of antioxidant enzymes had no significant change. In addition, in terms of male rats, high iodine intake decreased the SOD activity compared with the NI group (*U* = −2.192, *n* = 10, *p* = 0.029), but high-dose vitamin C significantly increased the activity of SOD, which was decreased by long-term excess iodine exposure (*U* = −2.030, *n* = 10, *p* = 0.043); however, the activities of TAOC (*U* = −2.041, *n* = 10, *p* = 0.043) and GSH-Px (*t* = 2.543, *df* = 19, *p* = 0.020) in the HI + HC group was lower than the NI group, and its CAT activity was lower than the HI (*U* = −2.117, *n* = 10, *p* = 0.035) and HI + LC (*U* = −2.192, *n* = 10, *p* = 0.029) groups. Thirdly, the female content of MDA was lower than that in males, but the difference was statistically significant only in the HI + HC (*U* = −2.684, *n* = 10, *p* = 0.006) group.

### 3.7. Protection of Vitamin C on Oxidative Damage in the Thyroid Caused by Excess Iodine

**Figure 5:**
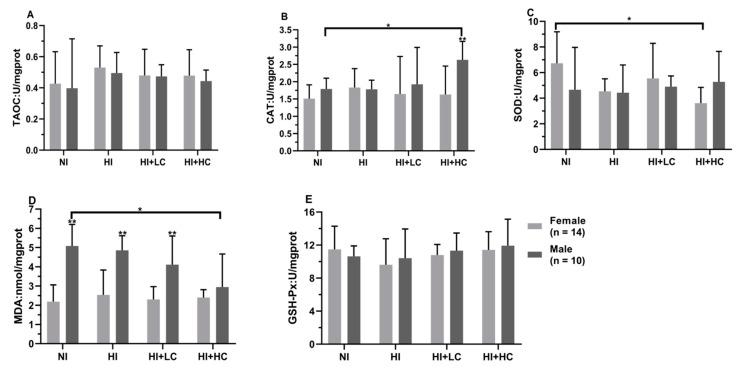
Protection of vitamin C from oxidative damage in the thyroid for different sexes. (**A**) The activity of TAOC; (**B**) The activity of CAT; (**C**) The activity of SOD; (**D**) The content of MDA; (**E**) The activity of GSH-Px. All data are presented as mean and deviation. ** represents the difference between female and male being statistically significant, and *p* < 0.01, and * represents *p* < 0.05.

The activity of CAT in NKI was statistically lower than that in NKIO_3_ (*U* = −2.619, *n* = 15, *p* = 0.008), Table 4. For KI, the CAT activity in the vitamin C intervention group was significantly higher (*H* = 8.574, *df* = 3, *p* = 0.036), which might be the result of body compensation, and long-term excess iodine exposure had effects on the GSH-Px activity and MDA content, but there was no statistical significance; in terms of KIO_3_, the activity of SOD in the NI and HI + LC groups was significantly higher than that in the HI and HI + HC groups (*H* = 8.353, *df* = 3, *p* = 0.039). In addition, from the perspective of different sexes, Figure 5, as for female rats, the SOD activity in the HI (*U* = −2.468, *n* = 20, *p* = 0.013) and HI + HC (*U* = −2.280, *n* = 20, *p* = 0.022) groups was significantly lower than the NI group, and then for male rats, compared with the NI and HI groups, high-dose vitamin C, respectively, increased and decreased the CAT activity (NI and HI + HC, *U* = −2.192, *n* = 10, *p* = 0.029; HI and HI + HC, *U* = −2.192, *n* = 10, *p* = 0.029) and the MDA content (NI and HI + HC, *U* = −2.041, *n* = 10, *p* = 0.043; HI and HI + HC, *U* = −2.192, *n* = 10, *p* = 0.029). Finally, from the comparison of female rats and male rats, the female content of MDA was lower than that in males except rats in the HI + HC (*U* = −5.178, *n* = 10, *p* = 0.000) group, and the activities of TAOC and GSH-Px had no statistical change among groups.

### 3.8. Protection of Vitamin C from Oxidative Damage in the Lens Caused by Excess Iodine

**Figure 6:**
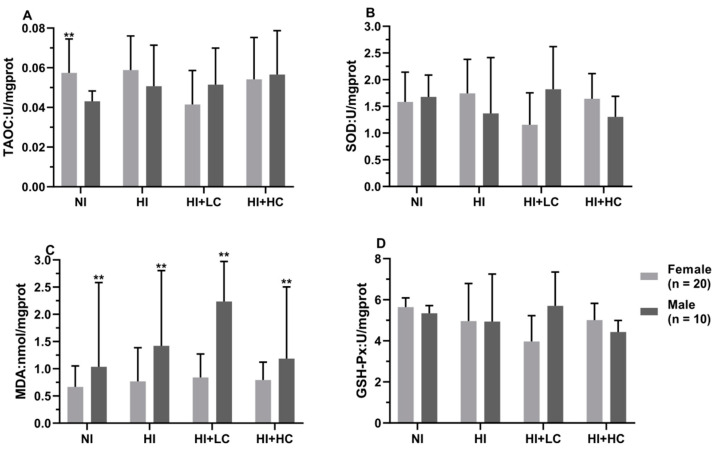
Protection of vitamin C from oxidative damage in the lens for different sexes. (**A**) The activity of TAOC; (**B**) The activity of CAT; (**C**) The activity of SOD; (**D**) The activity of GSH-Px. All data are presented as mean and deviation. ** represents the difference between female and male being statistically significant, and *p* < 0.01.

The changes in all antioxidant enzyme activities and MDA content in the lens were not statistically significant, Table S5, but it was found that the MDA content in each group of KI was higher than that of KIO_3_, and the MDA content increased in HKI + LC and HKIO_3_. In addition, the female content of MDA was significantly lower than that of males (Figure 6, *U* = −5.651, *n* = 10, *p* = 0.000), and the TAOC activity in the NI group also showed a sex difference: female was higher than male (*U* = −2.816, *n* = 10, *p* = 0.004). The activity of CAT in the lens was too low to determine.

## 4. Discussion

The ratio of sex was based on the sensitivity to oxidative damage. It was known that thyroid disease pathogenesis is related to oxidative stress; Rodrigo SF et al. found that compared with male rats, female rats showed higher mRNA expression of NOX4 and Poldip2 due to the influence of estrogen, which produced more hydrogen peroxide and decreased oxidation defense ability [33]. Therefore, it was reasonable to adopt a female and male ratio of 2:1 in this study.

Through this study, it was found that compared with KI, excessive intake of KIO_3_ decreased the serum activity of SOD, but there was no significant difference between the HI and NI groups, which was consistent with Wu YX et al. [34]. In addition, through a literature review, Zhang Y et al. found that the effect of KIO_3_ on the activities of antioxidant enzymes in experimental animals was greater than that of KI, but all the experimental doses far exceeded the doses obtained from salt [35].

Excess iodine intake could interfere with the production and activity of antioxidant enzymes by disturbing the synthesis of thyroid hormones [36]; moreover, iodine had the effect of antioxidation and promoting oxidation [37]. Compared with optimal iodine intake, it was found that the activities of antioxidant enzymes and the content of MDA in serum and tissues, respectively, decreased and increased with long-term excess iodine exposure in this experiment, which was consistent with the study by Xia Y et al. [38,39]). However, Joanta AE et al. found that excessive iodine intake could make the activity of antioxidant enzymes appear to compensatorily increase and lead to the increased content of MDA [40,41], and Malgorzata KL et al. thought that the body had a strong antioxidant defense system, and excess intake of KI or KIO_3_ would not lead to the change in lipid peroxide in tissue [42]. This discrepancy might be due to the variation in source, dose of iodine, and of rats. Moreover, there were differences in enzyme activities among organs; the enzyme activities in serum, the liver, and the kidney were higher than other tissues, especially in serum, which might be because excess iodine entering the stomach is first transported through the blood to the organs of the body, so the blood was exposed to high iodine earlier, and that the liver and kidney are the main organs of detoxification in the body.

The best way to prevent oxidative damage caused by high iodine is to reduce the intake of iodine, but the causes of high iodine could be attributed to the high iodine content of water, the diet, and iodized salt; among them, the high level of iodine in water and the diet is difficult to correct, and the high level of iodine in salt is also limited by the storage conditions of iodized salt. As a result, additional supplements of vitamin C are a better method to counteract the oxidative damage caused by excess iodine exposure. Vitamin C represents one of the most prominent antioxidants both in plasma as well as intracellular regions; enables the quenching and scavenging of free radicals; and is required in the body for collagen formation in the bones, blood vessels, and muscles [39]. Robert D et al. found that vitamin C could reduce oxidative stress in hemodialysis patients [43], and Chetanjyoti T et al. found that vitamin C could increase the activity of antioxidant enzymes in arsenic-induced oxidative damage [44]. In this study, it could be found that vitamin C can increase the activity of antioxidant enzymes, which decreased in the HI group, and had a protective effect on oxidative stress caused by excessive iodine. The improvement in the enzymatic activity of groups receiving vitamin-C-supplemented feeds could be due to the ability of ascorbic acid to debug ROS and RNS such as hydroperoxyl radicals, ozone, singlet oxygen, nitrogen dioxide, nitroxide radicals, etc., thereby preventing other substrates from oxidative damage in the cells [45]. However, compared with rats in the NI group, there was no significant change in antioxidant enzyme activity in the HI + LC group, but they had a high content of MDA in serum, the liver, and the kidney; it was higher than that in the HI group. This might be due to the promoting role of vitamin C in lipid peroxidation caused by the iron-induced Fenton reaction [46]. Hydrogen peroxide and the divalent iron ion are both substrates of the Fenton reaction [47], which might explain why the activity of CAT increased in the HI + LC group. Different from the effect of low vitamin C, high vitamin C could significantly reduce the level of lipid peroxidation, which was consistent with the results of Li ZL et al. [48]. While we also found that the activities of some antioxidant enzymes in the HI + HC group were lower than those in the NI group, which might be due to the fact that the enzymatic antioxidant system and the non-enzymatic antioxidant system (including vitamin C) complement each other, the supplementation of vitamin C would affect the enzymatic antioxidant system [49].

This study is of great significance in exploring the protection from oxidation damage caused by excess iodine intake. Firstly, due to the limitation of time, this study did not design an intervention with different times, and it was conducted in a later study; in addition, because of the sample size, the contents of vitamin C in the blood and organs were not determined in the study. If there is an opportunity in the future, we will expand the sample size of the rats, especially male rats, to further study the exact mechanism of vitamin C on oxidative damage caused by excess iodine exposure.

## 5. Conclusions

Long-term chronic excessive iodine exposure caused oxidative damage in rats, such as decreasing the activity of antioxidant enzymes and increasing the content of lipid peroxides, and there was a difference between females and males. Vitamin C had a certain protective effect against oxidative damage induced by excess iodine exposure; a high-dose intake of vitamin C reduced the content of MDA, while a low-dose intake of it promoted oxidative damage.

## Figures and Tables

**Table 1:** Urinary iodine.

Iodine Type	Classification	Different Sex (μg/L) M, (P_25_–P_75_)		Urinary Iodine M, (P_25_–P_75_) (*n* = 15, μg/L)	*p* (HI, HI + LC, and HI + HC)
		Female (*n* = 10)	Male (*n* = 5)		
KI	NI	74.98 (52.07–88.42)	70.14 (55.83–96.37)	73.53 (52.62–89.36)	
	HI	3371.40 ** (3163.89–5007.75)	3188.63 ** (2628.76–4145.06)	3334.50 ** (2388.63–4904.93)	0.183
	HI + LC	4363.17 ** (3132.34–5136.92)	4935.32 ** (3427.49–5914.56)	4540.89 ** (3263.55–4981.27)	
	HI + HC	3951.08 ** (2830.47–5167.89)	3424.45 ** (2460.64–4278.64)	3581.56 ** (2007.12–4663.44)	
KIO_3_	NI	68.50 (55.28–90.44)	64.20 (49.01–90.93)	67.18 (52.50–96.42)	
	HI	3592.30 ** (2710.815803.82)	4755.90 ** (3562.59–7044.47)	3662.14 ** (2984.43–5859.09)	0.840
	HI + LC	3910.33 ** (2668.45–5807.05)	3513.58 ** (2857.31–4001.91)	3656.47 ** (2776.23–4512.95)	
	HI + HC	4256.03 ** (2642.97–4991.33)	4035.67 ** (3264.24–6639.86)	3957.14 ** (3012.70–4904.93)	

Note: ** represents that compared with NI, the urinary iodine was higher and *p* value was lower than 0.01.

**Table 2:** Protection of vitamin C from oxidative damage in liver.

Indicators	Classification	KI $\left(\bar{\chi }\;\pm \;S\right)$ (*n* = 15)	KIO_3_ $\left(\bar{\chi }\;\pm \;S\right)$ (*n* = 15)	*p*
TAOC U/mgprot	NI	0.45 ± 0.04	0.47 ± 0.06	0.486
	HI	0.48 ± 0.08	0.45 ± 0.07	0.217
	HI + LC	0.46 ± 0.06	0.47 ± 0.09	0.902
	HI + HC	0.49 ± 0.05	0.51 ± 0.07	0.838
	*p*	0.242	0.113	
CAT U/mgprot	NI	26.93 ± 11.12	21.62 ± 10.43	0.189
	HI	22.60 ± 10.85	22.73 ± 12.24	0.976
	HI + LC	28.65 ± 9.20	23.52 ± 7.25	0.101
	HI + HC	25.08 ± 10.93	23.33 ± 8.52	0.628
	*p*	0.404	0.951	
SOD U/mgprot	NI	101.88 ± 17.58	106.65 ± 19.36	0.486
	HI	100.20 ± 15.77	103.96 ± 15.61	0.517
	HI + LC	97.59 ± 10.82	99.18 ± 11.35	0.698
	HI + HC	104.65 ± 12.57	104.96 ± 16.26	0.954
	*p*	0.542	0.575	
MDA nmol/mgprot	NI	0.86 ± 0.16	0.92 ± 0.16	0.512
	HI	0.89 ± 0.13	0.86 ± 0.10	0.217
	HI + LC	1.08 ± 0.24	1.09 ± 0.36	0.713
	HI + HC	0.96 ± 0.25	0.86 ± 0.09	0.174
	*p*	0.023 * HI + LC > NI, HI, HI + HC	0.058	
GSH-Px U/mgprot	NI	459.81 ± 133.67	444.52 ± 78.29	0.706
	HI	439.16 ± 89.81	472.77 ± 119.85	0.392
	HI + LC	430.91 ± 131.21	444.88 ± 107.96	0.753
	HI + HC	454.28 ± 105.77	454.78 ± 114.50	0.990
	*p*	0.905	0.886	

Note: * represents *p* value being lower than 0.05.

**Table 3:** Protection of vitamin C from oxidative damage in kidney.

Indicators	Classification	KI $\left(\bar{\chi }\;\pm \;S\right)$ (*n* = 15)	KIO_3_ $\left(\bar{\chi }\;\pm \;S\right)$ (*n* = 15)	*p*
TAOC U/mgprot	NI	0.50 ± 0.09	0.57 ± 0.11	0.061
	HI	0.55 ± 0.10	0.53 ± 0.08	0.653
	HI + LC	0.50 ± 0.09	0.50 ± 0.10	0.976
	HI + HC	0.55 ± 0.10	0.58 ± 0.09	0.482
	*p*	0.350	0.141	
CAT U/mgprot	NI	10.90 ± 3.17	10.15 ± 3.37	0.542
	HI	9.88 ± 3.64	9.75 ± 2.68	0.914
	HI + LC	9.78 ± 4.05	9.87 ± 1.99	0.922
	HI + HC	10.99 ± 3.64	11.69 ± 4.35	0.638
	*p*	0.790	0.367	
SOD U/mgprot	NI	200.75 ± 36.75	201.10 ± 42.52	0.981
	HI	202.37 ± 43.74	203.60 ± 37.72	0.935
	HI + LC	192.73 ± 27.71	189.76 ± 24.78	0.759
	HI + HC	192.18 ± 19.63	195.69 ± 33.05	0.726
	*p*	0.835	0.707	
MDA nmol/mgprot	NI	4.01 ± 2.71	3.76 ± 2.68	0.983
	HI	4.68 ± 2.94	3.30 ± 1.61	0.161
	HI + LC	5.94 ± 3.51	6.04 ± 3.13	0.653
	HI + HC	4.32 ± 2.82	3.85 ± 2.43	0.624
	*p*	0.015 * HI + LC > NI, HI, HI + HC	0.005 ** HI + LC > NI, HI, HI + HC	
GSH-Px U/mgprot	NI	214.62 ± 46.49	231.71 ± 44.08	0.319
	HI	216.04 ± 59.11	199.77 ± 59.77	0.450
	HI + LC	201.57 ± 47.57	211.36 ± 42.79	0.558
	HI + HC	210.20 ± 56.12	224.12 ± 42.96	0.452
	*p*	0.875	0.281	

Note: * represents *p* value being lower than 0.05; ** represents *p* value being lower than 0.01.

**Table 4:** Protection of vitamin C from oxidative damage in the thyroid.

Indicators	Classification	KI $\left(\bar{\chi }\;\pm \;S\right)$ (*n* = 12)	KIO_3_ $\left(\bar{\chi }\;\pm \;S\right)$ (*n* = 12)	*p*
TAOC U/mgprot	NI	0.47 ± 0.20	0.52 ± 0.22	0.486
	HI	0.58 ± 0.33	0.50 ± 0.13	0.838
	HI + LC	0.43 ± 0.14	0.53 ± 0.19	0.161
	HI + HC	0.46 ± 0.15	0.51 ± 0.14	0.345
	*p*	0.714	0.960	
CAT U/mgprot	NI	1.38 ± 0.31	1.96 ± 0.71	0.008 **
	HI	1.58 ± 0.68	2.08 ± 1.15	0.265
	HI + LC	1.91 ± 0.78	2.06 ± 0.81	0.806
	HI + HC	2.10 ± 0.74	2.16 ± 0.92	0.998
	*p*	0.036 * NI, HI < HI + LC, HI + HC	0.972	
SOD U/mgprot	NI	5.51 ± 2.92	6.75 ± 2.29	0.246
	HI	4.71 ± 2.10	4.39 ± 2.08	0.583
	HI + LC	5.14 ± 2.89	5.53 ± 2.23	0.533
	HI + HC	4.84 ± 2.36	5.03 ± 2.53	0.653
	*p*	0.967	0.039 * NI, HI + LC > HI, HI + HC	
MDA nmol/mgprot	NI	3.51 ± 2.27	2.85 ± 1.44	0.775
	HI	4.09 ± 1.85	3.08 ± 1.30	0.137
	HI + LC	2.77 ± 1.25	2.93 ± 1.59	0.806
	HI + HC	2.51 ± 1.16	3.04 ± 1.28	0.250
	*p*	0.105	0.958	
GSH-Px U/mgprot	NI	10.54 ± 2.66	11.72 ± 1.79	0.215
	HI	9.28 ± 3.20	10.62 ± 3.31	0.326
	HI + LC	11.37 ± 1.93	10.67 ± 9.91	0.309
	HI + HC	11.92 ± 2.41	11.34 ± 2.88	0.596
	*p*	0.074	0.696	

Note: * represents *p* value being lower than 0.05; ** represents *p* value being lower than 0.01.

## Data Availability

The data presented in this study are available on request from the corresponding author. Because the data involve information that has not yet been published, the data described in the manuscript, code book, and analytic code will not be made available.

## Supplementary Materials

The following supporting information can be downloaded at: https://www.mdpi.com/article/10.3390/nu14245245/s1, Table S1. Water consumption and iodine consumption; Table S2. Food consumption and iodine intake; Table S3. Protection of vitamin C from oxidative damage in serum; Table S4. Protection of vitamin C from oxidative damage in brain; Table S5. Protection of vitamin C from oxidative damage in lens.
