# Supplementary material for: Protection of Vitamin C on Oxidative Damage Caused by Long-Term Excess Iodine Exposure in Wistar Rats
Source: Nutrients. 2022 Dec 9;14(24):5245. doi: 10.3390/nu14245245 (PMC9786336; doi:10.3390/nu14245245)
Supplement: Supplementary file 1 [file nutrients-14-05245-s001.zip › nutrients-2020534-supplementary.pdf]

Table S1. Water consumption and iodine consumption

**Month**	**Classification**	**Male mL/w ($\bar{\chi} \pm S$)**		**Female mL/w ($\bar{\chi} \pm S$)**	
		**KI** (*n* = 4)	**KIO_3** (*n* = 4)	**KI** (*n* = 8)	**KIO_3** (*n* = 8)
First	NI	3008.25±134.29	2667.70±145.67	1914.22±143.31	1987.49±113.38
	HI	2767.19±163.02	3054.76±119.91	1846.28±166.81	1856.90±311.35
	HI+LC	2736.65±255.98	2427.71±165.58	1795.57±221.60	1499.38±157.29
	HI+HC	2819.26±465.82	2820.73±205.66	1801.99±284.11	2106.65±355.33
	P	0.556	0.001**<br>HI+LC<NI,HI+HC<HI	0.661	0.000**<br>HI+LC<HI,NI,HI+HC
Second	NI	2845.65±431.32	2864.85±629.72	1991.96±407.85	2031.28±202.30
	HI	2643.35±402.41	3003.13±583.71	1942.16±253.38	1861.61±395.72
	HI+LC	2784.55±535.82	2877.60±475.12	1816.90±232.91	1515.90±312.49
	HI+HC	2786.03±529.20	2575.80±435.69	1905.54±213.22	2153.34±300.09
	P	0.647	0.936	0.666	0.212
Third	NI	3350.25±449.06	3188.78±558.50	1951.15±412.59	2642.90±448.19
	HI	2956.20±569.24	3568.95±494.79	1891.65±530.69	2246.50±560.97
	HI+LC	3569.85±448.59	2902.73±587.47	2125.28±609.81	1536.70±100.89
	HI+HC	3242.30±525.05	3248.45±602.54	2057.25±320.81	2696.74±361.36
	P	0.411	0.450	0.322	0.059
Fourth	NI	3030.18±293.33	2957.60±147.17	2194.74±150.52	2261.53±166.49
	HI	2576.88±132.71	3412.95±333.58	2064.14±241.26	2109.28±381.42
	HI+LC	3318.43±336.20	2521.43±52.80	1858.68±214.39	1660.78±419.33
	HI+HC	2958.73±35.37	2968.08±76.40	2127.14±90.50	2387.04±67.29
	P	0.006**<br>HI<NI,HI+LC,HI+HC	0.000**<br>HI+LC<NI,HI+HC<HI	0.007**<br>HI+LC<NI,HI,HI+HC	0.001**<br>HI+LC<HI,NI,HI+HC

Note: ** represented that P value was lower than 0.01.

Table S2. Food consumption and iodine intake

Month	Classification	**Male** g/w (χ ± S)		**Female** g/w (χ ± S)	
		**KI** (*n* = 4)	**KIO_3** (*n* = 4)	**KI** (*n* = 8)	**KIO_3** (*n* = 8)
First	NI	902.82±16.33	896.83±9.33	689.69±26.55	696.12±30.76
	HI	911.60±20.18	917.27±29.08	702.42±45.61	662.68±58.32
	HI+LC	862.47±24.14	873.92±26.05	652.45±38.79	663.47±30.06
	HI+HC	884.91±6.34	898.51±44.79	650.07±21.39	710.74±16.47
	P	0.183	0.292	0.002**<br>NI,HI>HI+LC.HI+HC	0.039*<br>NI,HI+HC>HI,HI+LC
Second	NI	929.82±41.59	893.25±30.30	671.05±37.19	718.17±25.75
	HI	855.82±36.17	927.24±10.83	680.74±32.65	647.51±24.94
	HI+LC	858.01±43.19	863.34±29.18	646.15±24.04	647.86±18.44
	HI+HC	838.96±14.31	881.39±31.25	621.62±22.19	702.44±30.85
	P	0.017*<br>NI>HI,HI+LC,HI+HC	0.025*<br>NI,HI+LC,HI+HC<HI	0.000**<br>NI,HI>HI+LC,HI+HC	0.000**<br>NI,HI+HC>HI,HI+LC
Third	NI	891.83±21.85	830.95±9.73	631.53±28.26	644.11±45.87
	HI	795.83±22.90	824.80±7.40	625.69±36.54	610.10±32.06
	HI+LC	818.85±32.47	793.98±20.21	566.89±20.10	599.10±14.35
	HI+HC	859.35±10.98	837.08±57.04	630.84±22.52	675.71±30.04
	P	0.000**<br>NI,HI+HC>HI,HI+LC	0.255	0.002**<br>NI,HI,HI+HC>HI+LC	0.002**<br>NI,HI+HC>HI,HI+LC
Fourth	NI	910.53±33.06	870.45±24.32	670.56±31.84	675.40±37.78
	HI	842.05±21.12	887.45±16.78	659.53±42.48	632.40±34.60
	HI+LC	874.68±27.56	835.60±6.55	633.29±29.13	638.70±20.99
	HI+HC	881.10±26.79	894.30±9.49	681.29±16.54	733.78±26.31
	P	0.030*<br>NI,HI+LC,HI+HC>HI	0.001**<br>NI,HI,HI+HC>HI+LC	0.030*<br>NI,HI+HC>HI,HI+LC	0.000**<br>HI+HC>NI>HI,HI+LC
Iodine intake [a] (μg/d)	NI	17.35	16.64	12.39	12.95
	HI	3920.66	4669.71	2775.53	2892.62
	HI+LC	4444.20	3844.03	2721.93	2453.11
	HI+HC	4228.88	4190.04	2827.73	3347.08

Note: ** represented that P value was lower than 0.01, and * represented that $P<0.05$.

[a] represented the iodine intake of each day, which was calculated through water and food consumption and iodine amount of them (food, 500μg/kg), and because of the data size, the data variation was not displayed.

Table S3. Protection of vitamin C from oxidative damage in serum.

**Indicators**	**Classification**	**KI** ($\bar{\chi}$ ± S) (***n*** **= 15)**	**KIO₃** ($\bar{\chi}$ ± S) (***n*** **= 15)**	***P***
TAOC[a] U/mL	NI	4.03±1.21	3.85±1.00	0.671
	HI	3.95±1.04	4.12±1.55	0.741
	HI+LC	4.59±1.53	3.77±1.22	0.117
	HI+HC	4.68±1.18	4.14±1.00	0.199
	P	0.416	0.842	
CAT[b] U/mL	NI	3.39±1.24	4.06±1.49	0.178
	HI	3.62±1.48	3.85±2.21	0.967
	HI+LC	3.86±1.67	4.27±1.87	0.567
	HI+HC	3.09±1.45	3.23±1.17	0.591
	P	0.490	0.387	
SOD[c] U/mL	NI	267.36±17.43	259.36±42.55	0.769
	HI	268.23±36.70	235.32±47.64	0.005**
	HI+LC	257.63±43.83	258.49±44.41	0.806
	HI+HC	260.93±44.12	254.76±43.62	0.747
	P	0.622	0.192	
MDA[d] nmol/mL	NI	4.13±0.77	4.77±1.03	0.085
	HI	4.69±1.52	5.01±1.47	0.512
	HI+LC	4.82±1.35	4.72±1.55	0.775
	HI+HC	4.39±1.54	4.64±1.48	0.477
	P	0.341	0.831	
GSH-Px[e] U/mL	NI	1892.82±290.90	1925.41±380.45	0.946
	HI	1849.76±302.39	1890.22±454.37	0.512
	HI+LC	1854.59±297.15	1890.92±369.58	0.935
	HI+HC	1916.51±356.05	1780.27±202.89	0.451
	P	0.964	0.635	

Note: ** represented that P value was lower than 0.01.

[a] TAOC, total antioxidant capacity;

[b] CAT, Catalase;

[c] SOD, superoxide dismutase;

[d] MDA, malondialdehyde;

[e] GSH-Px, glutathione peroxidase;

Table S4. Protection of vitamin C fromoxidative damage in brain.

Indicators	Classification	KI ($\bar{\chi}$ ± S) (*n* = 15)	KIO_3 ($\bar{\chi}$ ± S) (*n* = 15)	*P*
TAOC U/mgprot	NI	0.22±0.11	0.26±0.10	0.285
	HI	0.21±0.10	0.21±0.06	0.567
	HI+LC	0.18±0.06	0.18±0.06	0.806
	HI+HC	0.18±0.09	0.20±0.11	0.870
	P	0.443	0.060	
CAT U/mgprot	NI	0.23±0.05	0.24±0.12	0.870
	HI	0.25±0.12	0.26±0.07	0.325
	HI+LC	0.25±0.10	0.23±0.10	0.512
	HI+HC	0.22±0.10	0.21±0.06	0.653
	P	0.732	0.391	
SOD U/mgprot	NI	74.45±11.97	80.75±13.68	0.325
	HI	70.87±10.30	76.85±18.52	0.512
	HI+LC	73.37±12.87	74.31±7.03	0.838
	HI+HC	74.51±13.92	73.76±10.54	0.806
	P	0.826	0.439	
MDA nmol/mgprot	NI	5.86±1.04	5.23±0.81	0.089
	HI	5.20±0.78	5.58±1.29	0.838
	HI+LC	5.31±1.00	5.39±1.19	0.935
	HI+HC	4.92±0.89	4.94±1.19	0.838
	P	0.101	0.635	
GSH-Px U/mgprot	NI	11.25±2.42	12.12±2.33	0.322
	HI	10.24±1.79	11.32±2.12	0.143
	HI+LC	10.52±1.44	10.84±2.60	0.682
	HI+HC	10.47±2.29	10.81±2.19	0.685
	P	0.527	0.413	

Table S5. Protection of vitamin C from oxidative damage in lens.

**Indicators**	**Classification**	**KI** ($\bar{\chi}$ ± S) (***n*** **= 15)**	**KIO_3** ($\bar{\chi}$ ± S) (***n*** **= 15)**	***P***
TAOC U/mgprot	NI	0.06±0.02	0.06±0.02	0.486
	HI	0.06±0.03	0.06±0.02	0.202
	HI+LC	0.06±0.03	0.05±0.02	0.267
	HI+HC	0.06±0.02	0.06±0.02	0.683
	P	0.602	0.055	
SOD U/mgprot	NI	1.55±0.78	1.66±0.55	0.567
	HI	1.73±0.80	1.88±0.90	0.683
	HI+LC	1.48±0.74	1.54±0.63	0.713
	HI+HC	1.53±0.61	1.61±0.55	0.744
	P	0.856	0.795	
MDA nmol/mgprot	NI	1.52±1.38	0.94±0.68	0.106
	HI	1.85±1.79	1.22±1.04	0.624
	HI+LC	1.65±0.98	1.55±1.24	0.250
	HI+HC	1.81±1.61	0.98±0.72	0.050
	P	0.626	0.242	
GSH-Px U/mgprot	NI	5.39±2.26	5.30±0.98	0.653
	HI	5.18±2.08	5.80±2.83	0.624
	HI+LC	4.97±2.46	4.88±1.43	0.624
	HI+HC	5.12±1.89	4.84±1.34	0.775
	P	0.851	0.486	
